# Robotic and laparoscopic surgery of the pancreas: an historical review

**DOI:** 10.1186/s42490-019-0001-4

**Published:** 2019-01-30

**Authors:** Alan Kawarai Lefor

**Affiliations:** Department of Surgery, Jichi Medical University, Shimotsuke, Tochigi Japan

**Keywords:** Pancreas, Cancer, Laparoscopy, Robotic surgery

## Abstract

Surgery of the pancreas is a relatively new field, with operative series appearing only in the last 50 years. Surgery of the pancreas is technically challenging. The entire field of general surgery changed radically in 1987 with the introduction of the laparoscopic cholecystectomy. Minimally Invasive surgical techniques rapidly became utilized worldwide for gallbladder surgery and were then adapted to other abdominal operations. These techniques are used regularly for surgery of the pancreas including distal pancreatectomy and pancreatoduodenectomy. The progression from open surgery to laparoscopy to robotic surgery has occurred for many operations including adrenalectomy, thyroidectomy, colon resection, prostatectomy, gastrectomy and others. Data to show a benefit to the patient are scarce for robotic surgery, although both laparoscopic and robotic surgery of the pancreas have been shown not to be inferior with regard to major operative and oncologic outcomes. While there were serious concerns when laparoscopy was first used in patients with malignancies, robotic surgery has been used in many benign and malignant conditions with no obvious deterioration of outcomes. Robotic surgery for malignancies of the pancreas is well accepted and expanding to more centers. The importance of centers of excellence, surgeon experience supported by a codified mastery-based training program and international registries is widely accepted. Robotic pancreatic surgery is associated with slightly decreased blood loss and decreased length of stay compared to open surgery. Major oncologic outcomes appear to have been preserved, with some studies showing higher rates of R0 resection and tumor-free margins. Patients with lesions of the pancreas should find a surgeon they trust and do not need to be concerned with the operative approach used for their resection. The step-wise approach that has characterized the growth in robotic surgery of the pancreas, in contradistinction to the frenzy that accompanied the introduction of laparoscopic cholecystectomy, has allowed the identification of areas for improvement, many of which lie at the junction of engineering and medical practice. Refinements in robotic surgery depend on a partnership between engineers and clinicians.

## Background

“Eat when you can,

Sleep when you can

Don’t mess with pancreas”

These succinct “three rules of surgery” represent how pancreatic surgery stands apart from other areas of General Surgery and the reverence (and fear) that generations of surgeons have had for this organ [1, 2]. Surgery of the pancreas (open, laparoscopic or robotic) is a technical challenge. The purpose of this review is to examine the role of robotic surgery as it is now practiced in the management of lesions of the pancreas. Robotic surgery is the third level of a three-story structure, with laparoscopic surgery as the second level, and everything built on a foundation of open surgery. We will use history as the guide as we ascend this three-story structure, starting with open surgery of the pancreas, then to laparoscopic surgery and laparoscopic surgery of the pancreas, then robots and robotic surgery and finally to robotic surgery of the pancreas. We need a vision of where we have been in this field to understand how we reached the point we are at today.

## Main text

### History of pancreatic surgery

Pancreatic surgery as we know it developed at the end of the nineteenth century. At that time, surgery for patients with obstructive jaundice was limited by coagulopathy, and palliative biliary bypass was developed to relieve obstruction caused by pancreatic malignancies [2]. These palliative bypasses originated in Russia and Switzerland, followed by Roux’s development of the Roux-en-Y bypass using a segment of intestine near the turn of the century. The next landmark in pancreatic surgery was the distal pancreatic resection. This portion of the gland was approached first because patients with these lesions were not jaundiced and there was less concern for coagulopathy.

In the early part of the twentieth century there were a number of surgeons who attempted and completed a variety of pancreatic resections but there was as yet no standardized approach to this organ. Surgeons performed isolated resection of carcinomas of the ampulla of Vater. Halstead did this in 1898 (a trans-duodenal approach), and through World War I there were three more isolated case reports of similar resections. Until about 1930, these four isolated cases represented the scope of surgery for malignancies in this region. This was indeed a rich era in surgical history, and the interested reader is invited to review the references used here and the references contained therein to obtain a detailed history of these procedures.

### Surgery for malignant lesions of the pancreas

The modern era of pancreatic resections for malignancies started in 1933 when Dr. Allen Oldfather Whipple, Chairman of the Department of Surgery at Columbia University College of Physicians and Surgeons (New York NY) invited Dr. Hap Mullins, a resident in the department, to develop the surgical technique for pancreatoduodenectomy (PD), known in the United States as the Whipple Operation, and in Japan as “PD”. After spending time in the laboratory, they performed a two-stage ampullary resection. Unfortunately, the patient died, possibly due to the use of catgut sutures in the pancreatic anastomosis. Whipple and Mullins persisted, changed the sutures to silk, and the second and third patients survived the surgery [2]. The pancreatic duct was ligated in these operations. Whipple’s first one stage resection was actually performed because of an error in the preoperative diagnosis [3]. During his career, Whipple performed the operation 37 times, with a mortality rate of about 33%. Pyloric preservation was introduced in 1968 by Longmire and Traverso, but the basic principles of the operation have not changed since its introduction by Whipple [2]. While some surgeons have attempted to modify the operation by performing more extensive resections such as total pancreatectomy, it is not clear that these operations resulted any survival advantages. Perioperative mortality rates changed little until the late twentieth century.

One of the major developments in the history of pancreatic surgery is the concept of Centers of Excellence, which routinely report postoperative mortality rates of < 2% [2]. One of the leading forces behind this change in practice originated at Johns Hopkins Medical Center in Baltimore MD under the leadership of Dr. John L. Cameron. By centralizing pancreatic resections in Maryland, it was shown that for every 1% increase in market share of PDs, in-hospital mortality decreased by 5% [3]. An impressive growth in case volume from 1970 to 2006 was associated with a reduction in mortality from 30 to 1%. This remarkable change was due to many contributing factors that came together to result in greatly improved patient outcomes.

### Minimally invasive surgery

As we trace the history of robotic surgery for malignant lesions of the pancreas, the next major historical milestone is the remarkable growth of laparoscopic surgery, which is one type of minimally invasive surgery. While it became popular among general surgeons starting in 1989, laparoscopic surgery had a long history by that time but was somewhat limited, being performed mostly by gynecologists. In the late 1980s, there was growing interest in the use of right upper quadrant mini-laparotomies for cholecystectomy. Mouret performed the first laparoscopic cholecystectomy in 1987, in France [4]. The operation was soon performed in the United States and the interest that exploded in this procedure was mirrored by the activity in the display area of the Clinical Congress of the American College of Surgeons in October 1989. The majority of these early procedures were performed at non-University medical centers, and only later did this approach become common at universities. One of the first laparoscopic cholecystectomies performed at a University medical center in the United States was at the University of Maryland Medical Center (also the origin of the widely used “Maryland Dissector”) in November 1989 by Karl Zucker, Robert Bailey and John “Jack” Flowers.

Early critics of the procedure suggested that it should be performed at specialized centers [5]. This was a true revolution in General Surgery and became unstoppable. One of the unique features of this revolution is that it did not start in academic laboratories. There was very little data to support or justify its use and the procedure rapidly spread throughout the world [5]. The financial benefits to the entire healthcare economy fueled the rapid growth of this entire field. Patients everywhere demanded that their operations were performed laparoscopically. The tools to perform the procedure were fairly new in 1987, especially the video-laparoscope and camera / display that allowed the surgical team to share the same view. Courses were held around the world to train surgeons in this new technique. There was suddenly no further interest in mini-laparotomy for cholecystectomy. As laparoscopic cholecystectomy became more widespread, there were many reports of bile duct injuries which raised significant concerns in the surgical and medico-legal communities. These seemed to be a result of the “learning curve” and are seldom discussed today as a particular consequence of using minimally invasive surgery techniques.

Within a few years, nearly every abdominal operation had been performed using minimally invasive surgery techniques. The techniques for abdominal minimally invasive surgery were rapidly adapted to minimally invasive surgery resections in the chest as well, such that thoracoscopic lung resections are the standard approach. The minimally invasive surgery approach is standard for operations such as appendectomy, Nissen fundoplication, colon resection, splenectomy, and others. There is further evolution going on in laparoscopic liver resection, laparoscopic gastrectomy, and other procedures.

As minimally invasive surgery techniques were adopted for the treatment of patients with malignancies, there were early reports of previously rare lesions such as port-site recurrences that raised many red flags in the surgical community. There were many questions raised about oncologic safety and long-term outcomes, and some of these remain unanswered, the majority have stood the test of time and study. The revolution in surgery created by the minimally invasive approach is nothing short of remarkable. It has resulted in improved patient outcomes, a wide range of changes in healthcare, and has fueled the rapid growth of many industries. It is not surprising that many people are searching for the “next revolution” in surgery.

### Laparoscopic surgery for malignant lesions of the pancreas

Despite the reverence (and fear) held by many surgeons regarding the pancreas, within a few years of the introduction of laparoscopic cholecystectomy, laparoscopic surgery of the pancreas had been attempted. The first laparoscopic PD was reported in 1994 [6]. Despite this early report of laparoscopic PD, the next series of developments in laparoscopic surgery of the pancreas related to distal pancreatectomy (DP). This is a less demanding technical procedure compared to PD, and laparoscopic DP has become a widely used approach for patients with benign or small malignant lesions of the distal pancreas [7]. Laparoscopic DP is the most widely used minimally invasive surgery approach to lesions of the pancreas. Many surgeons find that laparoscopic DP provides improved exposure and visualization compared with the open procedure, and patients have enhanced postoperative recovery with less morbidity [7].

The first large series of laparoscopic DP was reported in 1996 [8]. This was followed by a large number of comparative studies and meta-analyses [7, 9, 10]. Laparoscopic DP can be performed with or without splenic preservation. As of this writing, there have been no randomized controlled trials of laparoscopic DP vs. open DP [8, 9]. There was a meta-analysis of 12 non-randomized studies of laparoscopic DP reported in 2016 [9]. In aggregate, these studies included 1576 participants with 394 undergoing laparoscopic DP and 1182 undergoing open DP. The reviewers felt that the studies were of overall poor quality. There were no studies that examined quality of life outcomes. Overall, patients in the laparoscopic DP group had shorter hospital stays [9]. While laparoscopic DP is widely performed, there is no high-quality data to support this practice. Randomized prospective trials are needed to appropriately evaluate this application of minimally invasive surgery.

Some studies report a shorter hospital stay after laparoscopic DP compared with open DP [7]. Some also report decreased need for pain medication. In general, laparoscopic DP is associated with less intraoperative blood loss and longer operating times than the open DP. Mortality and morbidity rates of the two procedures are similar, as are the rates of pancreatic fistula formation. There is little data on long term oncologic outcomes. In summary, laparoscopic DP can be performed safely and effectively and has become the procedure of choice for lesions of the distal pancreas except in patients with large lesions or lesions in the central portion of the pancreas [7]. Given that laparoscopic DP is already the de facto standard, prospective trials may never be conducted, similar to what happened in the beginnings of laparoscopic cholecystectomy.

Although laparoscopic PD was first reported in 1994, large numbers of patients were not reported until much later. Cmpleting three anastomoses using minimally invasive surgery techniques is a technical challenge, which has limited the widespread adoption of this procedure. There have been quite a few series of laparoscopic PD reported, but there are no randomized trials to date. This operation can be performed safely. Some authors have reported a hybrid approach with mini-laparotomy or hand port [11]. In general, reviews have focused on indications, operative outcomes (e.g. blood loss, operative time, hospital stay) and short-term oncologic outcomes (e.g. lymph node resection) [7, 11].

Short-term outcomes in a small series from Japan were reviewed in 2009 [12]. These authors compared 15 patients who underwent laparoscopic PD from 2007 through 2008 with 15 patients who underwent open PD in the same time interval. The authors reported similar mean operative time and blood loss in both groups. The status of the surgical margins and numbers of lymph nodes were also similar, leading the authors to conclude that the two techniques have similar results.

In an unmatched retrospective review, Asbun reported 53 laparoscopic PD compared with 215 open PD and found significantly decreased blood loss and hospital stay in patients undergoing laparoscopic PD. [13] A meta-analysis of 12 comparative studies showed similar results with decreased blood loss and shorter hospital stay [14]. This meta-analysis also reported that some studies have shown higher mortality in patients undergoing laparoscopic PD compared with open PD, and this was attributed to procedures performed at low volume centers. The authors reported that five studies compared oncologic outcomes and one study reported that patients undergoing laparoscopic PD received adjuvant therapy earlier and had lower rates of local recurrence with overall survival similar to patients undergoing open PD.

A recent review specifically looked at short-term oncologic outcomes as well as overall survival [15]. The authors reviewed 828 patients who underwent laparoscopic PD and 7385 who underwent open PD from 2010 to 2013 using data from the National Cancer Data Base (US). The two groups were similar in terms of demographics and tumor characteristics. Using a multivariable model adjusted for hospital volume, laparoscopic PD was associated with a trend toward a shorter hospital stay (*p* < 0.14). The two groups had similar resection margin status, number of lymph nodes resected and perioperative mortality. Median overall survival was similar in the two groups.

Evaluation of laparoscopic PD by an expert panel is especially enlightening [14]. These experts concluded that laparoscopic PD is not a passing fancy but a technique that is here to stay. Training in this advanced procedure is essential. They feel it should be used as an approach in properly selected patients, and that intraoperative conversion to open surgery is not a complication. These experts also presented a list of “pros” and “cons” of both open and laparoscopic PD (Table 1).

**Table 1:** Advantages and disadvantages of laparoscopic and open PD (adapted from 14)

	Open	Minimally Invasive
Advantages	Standard and well-known	Reduced blood loss
	Operative Time	Reduced pain
	Operative Cost	Reduced wound complications
	Established training	Reduced hospital stay
	Tactile Feedback	More rapid recovery
		Magnified view
		Computer enhanced motion
Disadvantages	Blood loss	New approach
	Incisional pain	Increased operating time
	Wound morbidity	Equipment cost
	Hospital Stay	Loss of haptic feedback
	Recovery time	Lack of training opportunities

The role of high-volume centers in the conduct of laparoscopic PD has been examined [10]. A recent analysis of 7061 patients from the National Cancer Database showed that a majority of laparoscopic PDs were performed at low-volume centers, with less than 10 procedures per 2 years. This review found a significantly higher 30-day mortality rate compared with open PD although number of lymph nodes and status of surgical margins were similar. The authors describe a modularized training program for laparoscopic PD which includes four phases: Beginner (basic procedures and approach), Intermediate (Kocher maneuver, lesser sac, superior mesenteric vein tunnel), Advanced (dissection and division of major structures, anastomoses) and Expert (Pancreatic anastomosis). This defined teaching model may serve as a model for training in many other surgical techniques, particularly in robotic surgery.

As this procedure has become more widespread and less of a technical curiosity, investigators are focusing on complications associated with the procedure. Kantor and colleagues used data from the ACS-NSQIP data base [16]. Of 7907 patients undergoing PD, 1277 had PD performed using minimally invasive surgery approaches including 776 robotic or laparoscopic, 344 hybrid procedures and 197 unplanned conversions. Patients undergoing minimally invasive PD were less likely to have malignant lesions. The 30-day morbidity was less in the minimally invasive surgery group but 30-day mortality and length of stay were similar. They found a higher rate of postoperative pancreatic fistulas in the minimally invasive surgery group, but in conclusion they attribute this to case selection bias and do not feel it is inherent to the minimally invasive surgery approach. Dokmak and colleagues reviewed 46 laparoscopic and 46 open PDs performed at one center from 2011 to 14 [17]. They found that laparoscopic PD is associated with a significantly higher rate of pancreatic fistula. They conclude that laparoscopic PD should be limited to patients with a low risk of pancreatic fistula formation.

A survey was sent to the members of six international hepatobiliary surgical societies [18]. A total of 435 surgeons from 50 countries responded. Of these, 79% had performed laparoscopic DP and 29% had performed laparoscopic PD. The median personal experience was 20 cases of laparoscopic DP and 12 cases of laparoscopic PD. Respondents generally felt that laparoscopic DP is an important development but that laparoscopic PD needs further assessment. A lack of specific training was considered the major reason for not performing these procedures. Respondents would welcome an international registry. These results represent important opportunities for the future of laparoscopic and robotic pancreatic surgery.

Minimally invasive PD is now offered as a viable option in the care of patients with pancreatic malignancies in the guidelines of the National Cancer Control Network [19]. Further studies are needed to carefully evaluate long term outcomes. An international registry with standardized data collection would facilitate this. To date, available studies have not shown that outcomes are worse after laparoscopic PD, but they also have not shown any easily identified major advantages other than shorter hospital stay and decreased blood loss. While blood loss may be an important operative outcome, the need for transfusions may be of more clinical relevance, which has not been addressed to date. None of the studies have mentioned a comparison of hospital costs or charges in comparing open and laparoscopic PD. Last, attention is needed to assure appropriate training in this advanced procedure. These issues also must be addressed in the analysis of robotic PD, which is discussed extensively in the subsequent portion of this review.

### Robotic surgery

The word robot was coined by the Czech playwright Karel Capek (1890–1938) in 1920 for his play “Rossum’s Universal Robots”, commonly known as R.U.R., which premiered in Prague in 1921. Since that time, robots have permeated people’s imaginations, literature and factories. The word is derived from a Czech word which means “forced labor”. Robots are used in many facets of life, especially in manufacturing, greatly simplifying the production of many items, as well as allowing exploration of otherwise hazardous areas and other important applications. Robots are sure to play an even greater role in the future, largely made possible by rapid advances is sensing technology and computing on which the entire field of modern robotics is based. Given this, it is not surprising that the extensive use of robots in medicine was not possible until recent developments in microprocessor technology.

Surgical robotics actually has a fairly long history that became widespread soon after the widespread adoption of laparoscopic cholecystectomy. Before that time, there were some highly specialized robots used. The robotic approach to surgery is a direct outgrowth of laparoscopic surgery. The AESOP endoscope positioner was introduced in 1993, produced by Computer Motion Inc. (Santa Barbara CA), one of the first commercial entries in this field [20]. The DaVinci system was introduced in 1997 by Intuitive Surgical Inc. (Sunnyvale CA) and was cleared for use in the USA in 2000. The Zeus system was introduced by Computer Motion in 2001. Intuitive Surgical and Computer Motion subsequently merged. There are other robot systems in use and in development. At this time, the DaVinci system is the predominant robot used in surgical practice today. It is noteworthy that there was considerable initial interest by the military to conduct tele-robotic surgery close to the battlefield.

**Fig. 1:**
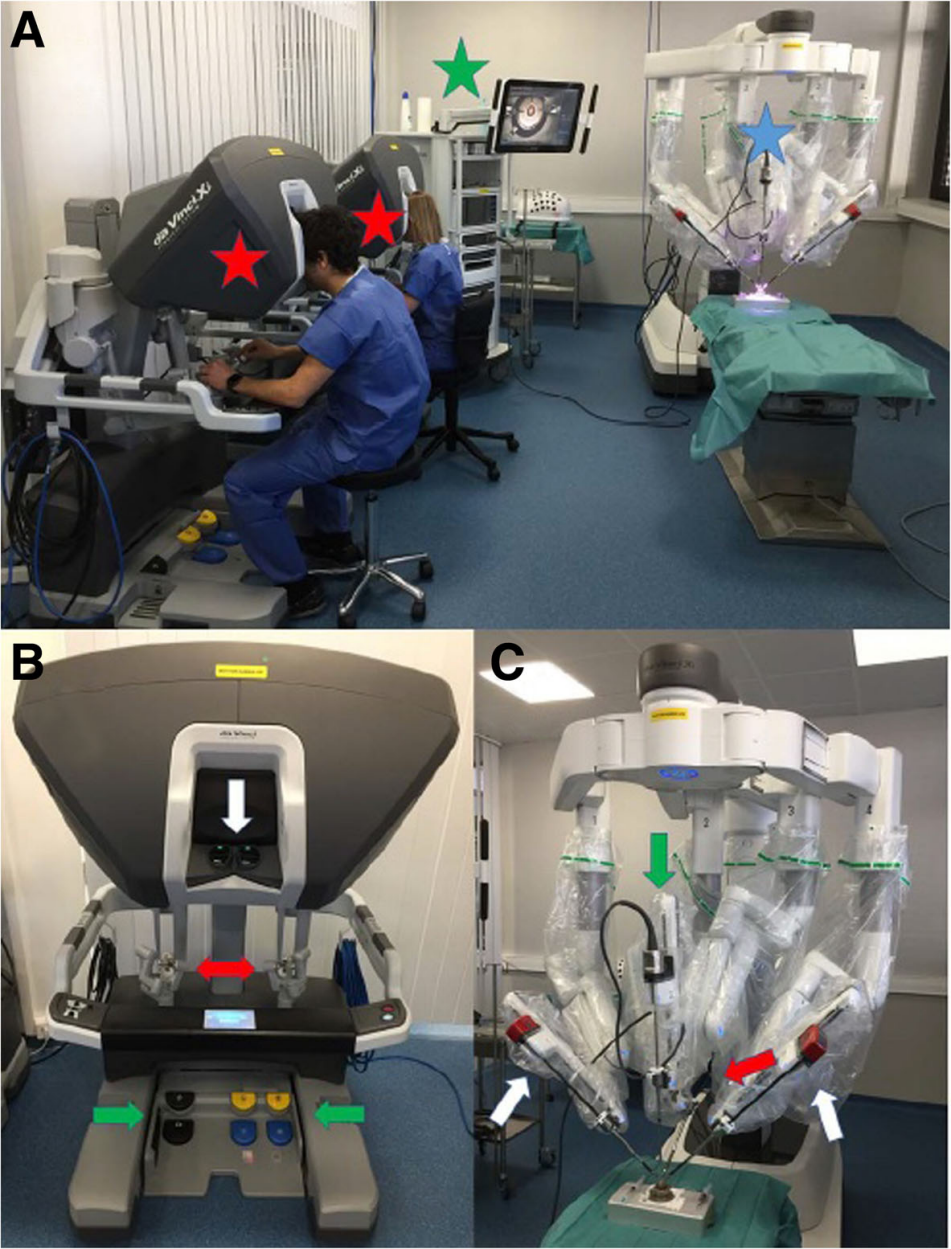
The DaVinci system includes three components, a patient cart, a surgeon console and a vision cart. **a** There are several patient carts available including the Xi (shown here), X and SP. The surgeon console and vision cart are shared among all models. The system shown here is for simulation and practice and includes two surgeon’s consoles. **b** The surgeon’s cart has an optical viewing system (white arrow), two manipulation handles (red arrows) and five pedals (green arrows). **c** The patient cart has the articulating arms which hold the instruments that are inserted into the patient. Reprinted under a Creative Commons license from Chammas J et al. Trans Vis Sci Tech 2017 6:21. doi: 10.1167/tvst.6.3.21

**Fig. 2:**
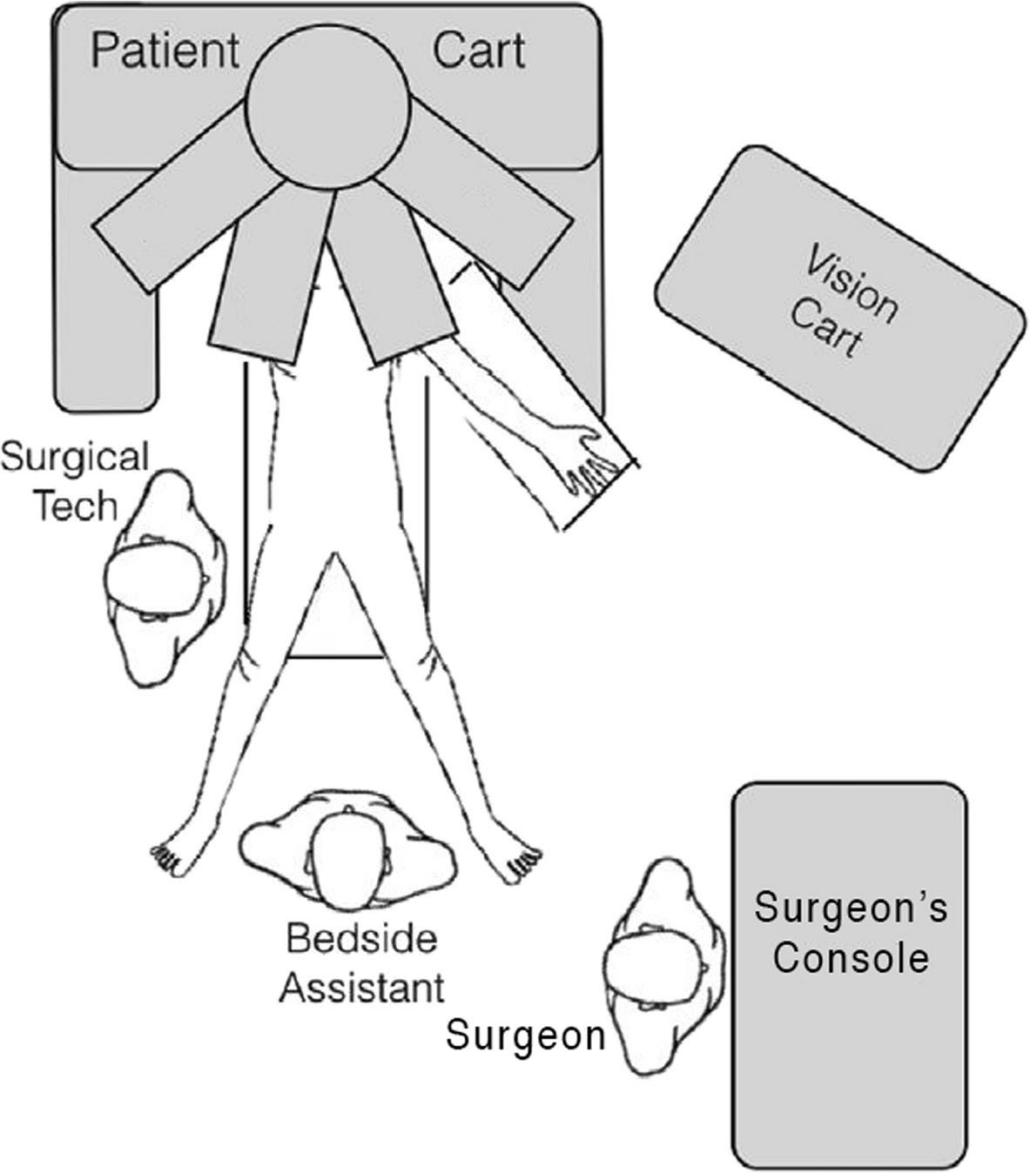
A typical configuration of the DaVinci robot in the operating room. Note that all team members have a clear view of the Vision Cart. Adapted from Ju YY and King JC. J. Vis Surgery 2017. 3:139. doi: 10.21037/jovs.2017.08.14

The DaVinci system is a master-slave system [21]. There are three main components including the patient cart, the surgeon’s console and the vision cart (Fig. 1). The instruments are inserted into the patient using similar methods as laparoscopic surgery then attached to the arms of the robot on the patient cart (Fig. 2). The surgeon sits at the surgeon’s console and manipulates the instruments using the robotic arms by moving controls at the console. The vision cart gives the same view to everyone in the operating room. Technically, this is robot-assisted surgery, since all motions are controlled by the surgeon’s hand. The tips of the instruments move in a manner determined by motion of the surgeon’s hands on the joysticks. The instruments move relative to the camera as the surgeon’s hands move relative to the eye. This enhances hand-eye coordination in robotic surgery. The system includes filtering of tremors, motion scaling and an internal articulated wrist. The DaVinci system costs approximately USD $2 M, as well as about $200,000 maintenance costs annually. There is a great deal of information available of various web sites for Intuitive Surgical [22, 23].

The use of robots in surgery is a natural extension of laparoscopic surgery. Nearly all operations that have been reported to have been performed robotically had already been reported laparoscopically. There is a seemingly natural progression from conventional open surgery to laparoscopic surgery and then to robotic surgery. The development of robotic surgery has been motivated by the related goals of overcoming the limitations associated with conventional laparoscopy as well as to further optimize outcomes [24]. Early in the development of surgical robots, the advantages of their use were clear [20]. Robot surgery provides three-dimensional visualization, improved dexterity and up to seven degrees of freedom. All of these are significant improvements over conventional laparoscopic surgery. Robots also standardize and smooth motion, eliminating tremors and scaling of motion. Their use allows tele-presence surgery which has already been performed from Europe to the USA [20]. The surgeon can sit in an ergonomic position, decreasing fatigue. Surgical robots simplify repetitive actions such as suturing deep in the pelvis.

There is a great deal of excitement in both the surgical community and by the public for the use of surgical robots. When laparoscopic cholecystectomy was initially becoming popular, many patients demanded that their surgeon perform “laser surgery”. Surgeons who did not perform laparoscopic cholecystectomy found many fewer referrals. This is happening to a degree regarding robot surgery, although not as commonly. Robot surgery definitely has associated concerns, in addition to the obvious questions regarding long-term efficacy when performed for the treatment of malignancies. For non-malignant conditions especially, there are significant concerns regarding increases in cost not only for the robot itself but for the longer time in the operating room. The start-up cost of using a robot is very high.

There is a wide range of terminology used in association with surgical robots including robotic surgery, robot-assisted surgery, robot-assisted laparoscopic surgery and so on. For the purpose of this review, all of these terms will be included under the single umbrella term robotic surgery. Nearly all robotic surgery today uses the master-slave system. The surgeon performs the surgery and is assisted by the robot [25] and this is sometimes referred to as robot-assisted surgery.

The requirements for robots in medical practice are different from those for industrial robots. Despite these rigorous requirements, robots are widely used in many areas of medical care and are used in virtually all surgical specialties. Diaz and colleagues made a careful analysis of the specific needs for robotics in medicine in order to guide future development [26]. They point out cost reduction, time of intervention, set-up time and complexity, reduced operating room footprint, data integration, and improved decision-making as clinical needs for robots in the future. They also carefully examine the technological possibilities that need to be matched to clinical needs as development advances in the future. Technical requirements that are highlighted include reduced size, shape and weight, increased numbers of degrees of freedom, reduced workspace needs, improved resolution in motion, platform stability, retraction of tissue, force (haptic) feedback, improved spatial orientation, wireless modules, triangulation, reduced need for instrument exchange, instrument flexibility, suctioning and irrigation, improved control requirements, improved ergonomics ad appropriate training. This paper is an excellent discussion of the interface between engineering and robotic surgery and points out many areas for future development.

### Training in robotic surgery

We are still in the early phases of development of robotic surgery. Perhaps as a result of the wild frenzy of adoption for laparoscopic surgery and the lack of a careful approach to training at that time, investigators and leaders in the field are developing educational programs as the field progresses. Some of this is being adopted from international working groups which are developing training in minimally invasive pancreatic resections in general, not limited to robotic surgery [27]. An international conference was held in 2016 in Brazil to focus on training and education issues. There is a definite learning curve associated with minimally invasive surgery of the pancreas, and low case volumes at many institutions make this a significant issue. Patient safety is not assured simply by surgical volume. The group concluded that a paradigm shift away from “see one, do one, teach one” is essential, and must be based on mastery of defined skills, including simulation and bio-tissue training. Centers of excellence must be developed to provide adequate training using a standardized approach and proctoring. Prospective reporting of patient data and outcomes must be part of the training program. Table 2 shows a credentialing pathway for advanced robotic hepato-pancreato-biliary surgery developed at the Beth Israel Deaconess Hospital in Boston MA (USA) [27].

**Table 2:** Credentialing pathway for Advanced Hepato-Pancreato-Biliary Surgery (adapted from 26)

• Step 1: Basic Preparation	
○ Didactic lectures, Online education	
○ Hands-on robotic training: docking, instrument exchanges	
○ Emergency Preparedness drills	
• Step 2: Advanced Preparation	
○ Robotic simulator training	
○ Procedure-specific video review	
• Step 3: Basic Robotic Hepato-Pancreato-Biliary Surgery	
○ Intraoperative mentoring by a credentialed robot hepato-pancreato-biliary surgeon	
○ 10 case minimum: cholecystectomy, wedge resection of the liver, partial gastrectomy	
• Step 4: Advanced Robotic Hepato-Pancreato-Biliary Surgery	
○ Intraoperative mentoring by credentialed robot hepato-pancreato-biliary surgeon	
○ Two-surgeon team until training is completed	

A recent study evaluated the learning curve for a single surgeon performing robotic PD. [28] This surgeon analyzed the results of 70 robotic PDs. There was one conversion to open surgery and one death within 30 days. There were postoperative complications in 75 and 10% of patients had complications with a Clavien-Davindo classification of Grade IIIb or worse. Operating time dropped significantly after 33 procedures, and there was a decrease in delayed gastric emptying as well after 33 procedures.

Formal training in robotic surgery has become common in the United States. In 2018, George and co-workers reported the results of a survey of general surgery program directors regarding training in robotic surgery [29]. Twenty program directors from medium sized programs were surveyed. Formal training in robotic surgery was conducted in 74% of programs, and 63% used simulation training. Most respondents felt that more time should be devoted to training in robotic surgery, and 63% felt that a formal program for training in robotic surgery should be part of the general surgery curriculum, including exposure in the first year of residency training. These results bode well for the future of training in robotic surgery which is essential to optimize outcomes.

Given the complexity of open surgery of the pancreas and the exponential increase in operative complexity with minimally invasive surgery (laparoscopy and robotic surgery), leaders in the field have correctly recognized the importance of adequate training. This is in contradistinction to what happened when laparoscopic cholecystectomy became widely performed, and there was little attention paid to formal training or performance benchmarks. Robotic surgery for malignancies of the pancreas in the Netherlands started in 2012 and was preceded by a great deal of preparation which is well documented [24]. First, they concluded that this should only be performed in high volume centers by an experienced operating team. All of the surgeons in this project were already experienced in pancreatic surgery, including open and conventional laparoscopic procedures. Operating room nurses and anesthesiologists were similarly experienced in pancreatic surgery. In the Netherlands, there was already a program for laparoscopic training at two levels, LAELAPS-1 and -2. To this was added LAELAPS-3 to give specific training in robotic surgery of the pancreas. The program in the Netherlands was developed in part through close collaboration with the University of Pittsburgh (Pittsburgh PA) [30] which already had initiated such specialty training. This training program includes a great deal of simulation training and training in specific surgical procedures such as suturing. The simulation training includes three phases: pre-test, curriculum and post-test. Training robots and artificial tissue are extensively. After successful completion of the training, the first clinical procedure is planned and includes careful patient selection and proctoring by an experienced robotic surgeon. This group identifies team work as the essential ingredient for success.

Surgeons from the University of Illinois – Chicago analyzed their experience over 15 years with more than 150 robotic PDs [19]. After carefully evaluating the operative procedure they distill the operation down to 17 essential steps. Each step is carefully described along with appropriate “tips and tricks”. Standardizing the steps of the procedure facilitates teaching it, and over time may result in improved results. The use of such a standardized approach should become an integral part of any randomized trials.

### Non-pancreatic robotic surgery

Before reviewing the current status of robotic surgery of the pancreas, we will briefly examine the literature regarding non-pancreatic surgery. This section of the review is not intended to be a detailed review of any one type of robotic surgery, nor is it in any way intended to be a meta-analysis. The purpose of this section is to provide a very broad overview of the field of robotic surgery. This is the view from the altitude of the International Space Station, not even the view at 40,000 ft. For readers interested in a more close-up view of the whole field may want to read an evidence-based report of the entire field as of 2012 [31]. While this report is somewhat dated, it does review available evidence for many types of robotic surgery.

When laparoscopic surgery was introduced to general surgery, most surgeons performed only laparoscopic cholecystectomy. It took a few years for this to widen to include other procedures. Perhaps because robotic surgery is not so radically different from laparoscopic surgery, robotic surgery has evolved fairly quickly to address many organs and compartments of the body. There is no one operation or organ that was favored as this field originated and expanded. We review a wide range of operations that have been performed robotically. All of these operations had been approached laparoscopically before taking the next step to robotic surgery.

The particular role of robotic surgery in the treatment of patients with malignancies has been described [25]. The authors speculate that robotic surgery may allow the conduct of more sophisticated procedures given the improved vision and dexterity offered by the robot. This includes more accurate resection margins and better lymph node resections. Whether this translates to improved clinical outcomes remains to be shown.

While this review is focused on robotic treatment of malignancies, there has been extensive experience with robotic surgery for bariatric procedures, although laparoscopic procedures remain the standard in this field. Laparoscopic bariatric surgery has been associated with relatively high complication rates. It is hoped that the improved dexterity associated with robotic surgery may decrease the rate of complications. This is worthwhile to review, if only because of the extensive worldwide experience in this area. Bariatric procedures are extremely common due to the rapidly increasing incidence of obesity throughout the world. Robotic Roux-en-Y gastric bypass was first reported in 1999 [32]. Early papers showed good outcomes and suggested a learning curve of about 10–15 procedures. The learning curve for the robotic procedure was shown to be less than for the laparoscopic procedure. In the laparoscopic bypass procedure, the anastomoses are generally performed with a stapler but many surgeons use a sutured anastomosis with the robot because of enhanced suturing ability made possible by the robot [21]. A meta-analysis of laparoscopic versus robotic bariatric surgery has been reported [32]. The authors identified 14 comparative studies, and found great heterogeneity in operative details. These authors note the change from stapled to sewn anastomoses and found a decreased leak rate in the robotic sewn anastomoses in some studies. Conversion rates are lower in some robotic series, but this can be attributed to the learning curve. Most surgeons performing the robotic procedure already have extensive experience with the laparoscopic procedure. Some studies reported a lower rate of postoperative strictures after the robotic procedure. Most studies in this meta-analysis found longer operating times with the robotic procedure. The low-level of evidence in the studies reviewed reinforces the need for improved study methodologies.

Robotic surgery has been used extensively outside of General Surgery. There have been a number of papers published describing robotic surgery of the head and neck. Robotic surgery of the pharynx, larynx, nasopharynx, sinuses, and anterior skull base have been described [33]. Radical neck dissections have also been performed robotically. The authors detail a large number of clinical trials in head and neck robotic surgery. Most of these studies are non-randomized. These authors discuss cost analyses and found that costs of laryngeal surgery performed robotically are 90% higher than conventional surgery. A detailed analysis shows that this is mostly due to the greatly increased cost of instrumentation.

Robotic surgery of the thyroid has been extensively described. Lee and colleagues conducted a careful study of the learning curve associated with robotic thyroid surgery [34]. This group began performing robotic thyroidectomy in 2007. This was a prospective multi-center study involving four endocrine surgeons at three centers. A total of 644 thyroid resections were evaluated. They compared results according to surgeon experience and determined that the learning curve for total thyroidectomy is 50 cases and for subtotal thyroidectomy it is 40 cases.

Robotic prostatectomy for cancer has received a great deal of attention in the last few years. It is very common for patients to demand this approach when they are told that they need resection. Yet, the data supporting robotic surgery for cancer of the prostate does not show a clear benefit in all studies. There have been few randomized prospective trials in this area. In a randomized prospective trial to examine short-term outcomes, investigators found similar functional outcomes comparing open and robotic radical prostatectomy [35]. There were benefits in the robotic group regarding less bleeding, fewer adverse events, earlier hospital discharge, and improved early postoperative quality of life. These investigators then followed the patients and reported long-term oncologic outcomes [36]. This study concludes that robotic surgery has functional outcomes equivalent to open surgery at 24 months. They caution that a lack of standardization in postoperative management may affect the results. They conclude that the benefits of a robotic resection are related to its minimally invasive nature. In a commentary regarding these studies, the senior author concluded that patients should choose a surgeon they trust, rather than making a decision based on the surgical approach [37].

A single center study of 31 patients who underwent robotic adrenal resection were compared with 31 consecutive patients who underwent laparoscopic resection [38]. When the data for all patients was analyzed the results were similar but when data for the last 20 patients in the robotic surgery group were analyzed separately (beyond the learning curve), the surgery in the robotic group was significantly shorter (139 vs 167 min, *p* < .05). Immediate postoperative pain was also less in the robotic surgery group.

Robotic distal gastrectomy for gastric cancer has also been reported. The use of this approach has been increasing rapidly in the last few years. A comparative study was reported comparing 109 patients who underwent robotic distal gastrectomy with 160 patients who underwent laparoscopic distal gastrectomy in the same time period [39]. The lesions were all stage cT1, and other patient characteristics were also similar in the two groups. They found a tendency (*p* = 0.112) toward reduced infectious complications in the robotic group. Injuries to the tail of the pancreas are well described in gastric surgery, and these injuries can result in a leak of amylase from the pancreas. The authors found significantly decreased amylase levels in the drains in patients in the robotic group, although this does not necessarily mean that clinically significant injuries to the pancreas occurred. At the very least, this study shows that robotic distal gastrectomy is comparable to laparoscopic surgery. Definitive randomized prospective trials are still lacking.

Robotic resection of colon cancer is well-described. One of the new approaches is to perform the resection through a single port to further reduce postoperative discomfort at port sites and also provide a superior cosmetic result. This has been done using laparoscopic surgical techniques and is now being used with robotic surgery. A meta-analysis of single port surgery for colon cancer has recently been reported [40]. Current studies show that single port robotic colon surgery is safe and feasible, but the quality of evidence in studies performed to date is low. The authors conclude that further advancements in robotic technology are needed to facilitate robotic single-port surgery.

This brief overview of non-pancreatic robotic surgery shows the breadth of surgery being approached with robotic techniques. When laparoscopic surgery was first used for the treatment of malignancies, there was a great deal of concern that there were unique risks inherent in this technique. Initially, this was borne out by reports of unusual complications such as port site metastases. Over time, these concerns have been alleviated and laparoscopic and robotic techniques are applied freely for the treatment of patients with malignancies.

### Robotic surgery of the pancreas

Pancreatic surgery for mass lesions is usually categorized as enucleation, DP or PD. Early robotic surgery of the pancreas was for the resection of benign lesions, and therefore we will first review this subject. Many benign lesions of the pancreas are resected by enucleation, and some potentially malignant lesions are similarly managed such as insulinomas of which 80% are benign. Robotic enucleation of pancreatic lesions has been reported [41]. The authors reported a series of five patients who underwent enucleation of lesions < 2 cm in the head (*n* = 2) and tail (*n* = 3) of the pancreas. The mean operative time was 204 min and mean blood loss 50 mL. They conclude that robotic enucleation is safe and feasible. This needs further study.

There have been a large number of studies of robotic DP, partly because this procedure is performed fairly commonly and because it is amenable to laparoscopic or robotic resection. It is less technically demanding than some other procedures, requiring minimal dissection and no reconstruction. Preservation of the splenic vein is technically challenging. This was first performed by Melvin in 2003 [42]. A recent meta-analysis compared robotic and laparoscopic distal pancreatectomy, and reviewed nine studies with 637 patients (246 robotic and 391 laparoscopic) [43]. The robotic procedure had an average stay one day shorter than the laparoscopic procedure, but the laparoscopic procedure was completed an average of 30 min shorter. They found no differences in feasibility, safety and oncologic adequacy. Another meta-analysis compared the laparoscopic and robotic procedures in 813 patients from ten studies [41]. There were no randomized controlled trials to evaluate. The studies included 267 patients resected robotically and 546 resected laparoscopically. The robotic group had a higher rate of spleen preservation, a lower rate of conversion to open surgery and a shorter hospital stay, but higher cost. The outcomes were similar in the two groups. Another meta-analysis reviewed nine studies with 238 patients resected robotically and 929 resected laparoscopically [44]. Four of the studies reported operative time, and there was no significant difference. There were also no differences in conversion to open surgery, spleen preservation rate, blood transfusion rate, pancreatic fistula rate or length of hospital stay. They concluded that robotic resection is safe, but that randomized controlled trials are needed. Another pooled analysis used data for 1815 patients from the ACS-NSQIP database to compare open, laparoscopic and robotic DP [45]. The series included 921 open procedures, 694 laparoscopic and 200 robotic DPs. The patients in the robotic group had longer operations and shorter hospital stays than the open group. Robotic resections took more time than laparoscopic resections, with fewer conversions to open. The authors conclude that each procedure offers advantages for well-selected patients, but demonstrating the most suitable use remains a challenge.

There are also a number of reports of DP from single institutions. From 2000 to 2013, 805 distal pancreatectomies were performed at Memorial Sloan-Kettering Cancer Center (New York NY) [46]. This included 37 robotic, 131 laparoscopic and 637 open procedures. Demographic characteristics were similar in the three groups. Pancreatic fistula rate and 90-day morbidity and mortality were similar in all three groups. Patients in the open surgery group were older, with a higher blood loss and a trend toward longer hospital stay. Oncologic outcomes were similar in the three groups. Both robotic and laparoscopic resections were similar with advantages over open resection in selected patients. Another study compared 102 patients undergoing robotic resection with 102 patients undergoing laparoscopic resection [47]. The robotic approach was associated with a lower rate of conversion to open surgery, improved spleen and splenic vein preservation and reduced hospital stay. All minimally invasive DPs from the University of Pittsburgh from 2004 to 2011 were compared, which included the first 30 robotic resections at that institution and 94 historical control laparoscopic resections [48]. Demographic variables were similar in the two groups. Postoperative length of stay, transfusion rate and readmission rates were similar in the two groups. Robotic resection reduced the rate of conversion to open surgery, and reduced the risk of excess blood loss. The robotic group had superior oncologic outcomes with a higher rate of negative margins and improved lymph node yield.

In an attempt to make a minimally invasive operation even less invasive, Kim and colleagues reported DP using two ports which the authors refer to as “single-site plus one port” [49]. This is an interesting report of six robotic distal pancreatectomies performed for a mass in the distal pancreas. The DaVinci single site platform was used with one additional port. The median operative time was 165 min with minimal blood loss. The indications for this procedure may expand, but are heavily dependent on operator experience as this would seem to be a highly technically demanding approach.

The first robotic PD was reported in 2001 by Giulianotti [19]. Since that time there have been many reports of this procedure and comparisons with laparoscopic PD. The technical demands of this procedure are formidable, both in regard to dissection and reconstruction of the biliary-enteric tract. The results of robotic PD have been reported to be generally similar to laparoscopic PD. [50] Operative times tend to be longer for the robotic procedure, while operative times for both laparoscopic and robotic PD are longer than for open PD. In a summary of robotic PD, while robotic surgery offers a stable platform, three-dimensional vision, and enhanced control of instruments, the effect of these features on overall outcomes is hard to show when compared to the laparoscopic procedure [50]. The lack of haptic feedback in robotic surgery remains a considerable drawback. These authors conclude that the main advantage of robotic surgery is centered on the surgeon, and not the patient. A systematic review of 13 studies representing 207 patients was reported [51]. The authors acknowledge the heterogeneity of the data, multiple definitions of robotic PD and wide range of options used for reconstruction. The morbidity was 58% and the reoperation rate was 7%. The authors conclude that robotic PD is feasible, with a wide range of surgical details and outcomes. In a systematic review, seven studies of robotic PD were analyzed [52]. Three of the studies were retrospective and four were prospective. Operative time ranged from 410 to 491 min, and 83% of patients had malignancies. Blood loss ranged from 100 to 634 mL, postoperative complications in 29 to 68%, mortality from 0 to 7%, an R0 resection in 73 to 100% and from 13 to 32 lymph nodes retrieved. There were four studies that compared open and robotic PD. The robotic PD was associated with less blood loss and a shorter hospital stay. The operative time for the robotic procedure was greatly impacted by the set-up time needed for the robot. The authors found a higher rate of R0 resections in the robotic group.

Boggi and colleagues reported a series of 200 robotic pancreatic resections, evaluated retrospectively [53]. The conversion rate to open surgery was 1.5%. PD was performed in 83 patients. Complications occurred in 63% and the reoperation rate was 7%. They compared to a contemporary group of open PDs and found that robotic PD took significantly longer in the operating room, with a similar safety profile, number of resected lymph nodes and positive resection margins for both procedures.

Robotic total pancreatectomy has also been reported. In a video case report of a patient with an intrapancreatic medullary neoplasm, Konstantinidis and colleagues present a succinct 16 step procedure for the conduct of a robotic total pancreatectomy [54]. In a review of data from the National Cancer Data Base, they evaluated the results of robotic total pancreatectomy in 73 patients and found similar rates of negative resection margins and number of lymph nodes resected compared with laparoscopic and open total pancreatectomy. The laparoscopic and robotic procedures were associated with shorter hospital stays and reduced operative mortality.

To further expand the use of robotic PD, a combined robotic PD and rectal resection for a patient with two malignancies was reported [55]. The authors tout the advantages of robotic surgery including three-dimensional vision, dexterity and ergonomics. While such operations are unlikely to performed by most surgeons, it shows what is possible.

Since robotic PD has become accepted, investigators are looking at other aspects of the procedure. One of the most common postoperative complications of pancreas surgery is a pancreatic fistula. A reduction in the incidence of postoperative fistulas is a major factor to improve overall morbidity associated with pancreas surgery [56]. McMillan and colleagues conducted a noninferiority study comparing robotic PD to open PD to determine the rate of clinically relevant pancreatic fistula occurrence [57]. This was a propensity score-matched analysis of 304 patients, and showed that robotic PD has a similar rate of clinically relevant pancreatic fistulas to open PD, and furthermore that robotic PD was non-inferior in terms of the occurrence of any complication, severe complications, hospital stay, 30-day readmission and 90-day mortality. This is an important study, supporting the conduct of robotic PDs. Robotic PD is not associated with an increased rate of fistulas. In another assessment of postoperative fistulas, Napoli and colleagues used a clinical risk score and identification of other factors predictive of postoperative fistulas [58]. Patients undergoing robotic PD and open PD were stratified into risk categories and matched by propensity scores. The authors found that in patients at intermediate risk of a fistula, robotic PD is associated with a higher rate of fistula after surgery. The rate of fistula formation was similar in the high-risk group. The overall morbidity and mortality were equivalent in the matched study groups. Importantly, these authors also performed a power analysis showing that the sample size for a non-inferiority randomized prospective trial would require 31,669 PDs to randomize 682 intermediate risk and 1852 high-risk patients. These numbers demonstrate that it is highly unlikely that a randomized trial can ever be conducted, and that registries will be needed to obtain useful data.

The complexity of robotic surgery of the pancreas has led to relatively slow adoption of the procedure on the world-wide scale, which has the benefit of allowing detailed analysis and appropriate emphasis on details as the procedure becomes more commonly performed. Patti and colleagues performed a value-based assessment of robotic pancreas and liver surgery [7]. They conducted a detailed analysis incorporating the interests of all groups involved. They review five series of robotic DP which also included cost analyses. For DP, one study found no significant differences in total costs for robotic, laparoscopic and open DP. Although robotic surgery has increased direct costs, there were net cost savings by reductions in length of stay. They review other studies which show that robotic surgery is significantly more expensive. The existing data is conflicting for robotic DP. In their attempt to review the costs of robotic PD, they conclude that there is insufficient data.

As robotic surgery moves forward, analyses of results will be dependent on the ability to identify appropriate metrics of effectiveness and quality of care. This was evaluated by Bassi and Andrianello, who emphasize the importance of considering all quality of indicators to ensure a high level of clinical care [55]. This is essential at all steps of patient care including assuring appropriate indications for the procedure, lowering the effects of morbidity by early recognition of adverse events, prevention of predictable complications, high standards of oncologic care and reduction of costs. They point out the positive effect of integrating minimally invasive pancreatic surgery with a dedicated team to monitor these important factors. Attention to these metrics will be beneficial as new centers of excellence are developed.

Given the expanding number of centers performing these operations, another area for standardization is the terminology associated with minimally invasive pancreatic resection. Montagnini et al. discuss how the heterogeneity on terminology leads to confusion and inconsistency [59]. They used a Delphi approach to develop a systematic terminology template that is an open structure which can accommodate future developments. This template combines the name of the operative approach and resection, taking into account the completion. It accounts for combined approaches as well.

It is clear from this rather superficial but broad review of available literature on robotic surgery of the pancreas, that there is still a dearth of quality data available regarding many aspects of these procedures. Future decisions regarding the use of robotic surgery for lesions of the pancreas should be based on data, but this is not possible today because there is not enough data. Furthermore, the collection of this data through high quality randomized controlled studies may not be possible for a wide range of reasons. Problems associated with research in this field were analyzed in detail by Barkun et al. [60]. Non-randomized studies may have to be depended upon for data. The authors discuss the development of a quality improvement program, which may greatly benefit the field of robotic surgery of the pancreas. Finally, they also emphasize the need for an international registry of robotic surgery of the pancreas. Robotic surgery of the pancreas represents a huge number of challenges and opportunities.

## Conclusions: What does this mean?

### For the surgeon

This has been an historical review, starting with the origins of pancreatic surgery, through the development of laparoscopic surgery including its applications in the treatment of patients with malignancies of the pancreas, the development of robotic surgery and finally to the use of robotic surgery in the treatment of patients with malignancies of the pancreas. This extremely broad review covers developments which took place only in the last 30 years or so, after the widespread adoption of laparoscopic cholecystectomy. At present, most studies find that robotic surgery for malignancies of the pancreas result in slightly shorter hospital stay and less blood loss. Some studies show a higher rate of R0 resection, and a higher rate of splenic vein preservation in DP with the robotic approach.

Conclusions are made more complex because of the heterogeneity of data collected. Randomized prospective trials are underway but the data has not yet been reported. These trials will be exceedingly difficult to complete and be adequately powered to give meaningful results for a variety of reasons. There are few reports of comparative costs of the procedure. We need data from multiple centers collected through international registries in a standard manner and we need adequate training programs to teach these advanced techniques. At the present time, the advantages of robotic surgery over laparoscopic surgery are centered on the surgeon and not the patient. Minimally invasive surgery (laparoscopic and robotic) approaches to malignancies of the pancreas are evolving techniques which will be further advanced by the efforts of investigators throughout the world.

### For the biomedical engineer

There are many areas in robotic surgery that require improvements that can only be made with a team effort including surgeons and Biomedical Engineers. Biomedical Engineers will have to understand what surgeons want, and the surgeons must understand what is possible with current limitations in technology. Some of the technologic challenges that have been identified include an increased number of degrees of freedom. Redundant motion with seven or more degrees of freedom may allow a more flexible arrangement of equipment [26]. Increased resolution of instrument motion will also benefit the surgeons. The lack of adequate haptic feedback has long been an issue identified in routine laparoscopic surgery and is even more lacking in robotic surgery where the surgeon’s hands do not hold the instruments. Improved visualization is always desirable, even with the implementation of three dimensional high-definition imaging systems. Improved control interfaces will facilitate the conduct of robotic procedures. Greater data integration will be helpful, allowing surgeons to view imaging studies in real time, with an augmented reality combined view. As technical metrics are developed for robotic surgery, it is essential that Biomedical Engineers are an integral part of the process. This discussion of areas for improvement is by no means complete, but merely an attempt to start the conversation.

### For the individual patient

We need to consider what this data means for an individual patient, who perhaps just found out that they have a pancreatic malignancy. They want the best possible treatment to maximize their chances for long-term survival. How can they use this data to achieve their personal goal as a patient? It is the conclusion of this author that the patient should find a hospital and a surgeon with experience, in whatever technique they use. Whether the operation is performed open, laparoscopically, or with a robot will likely not affect the long-term oncologic outcomes. This is good news for the patient, and what they are most concerned with.

Of these three approaches to the resection of malignant lesions of the pancreas, none is a “clear winner” or “clear loser”. Patients should expect their surgeon to use the technique with which they are most adept. If the procedure is performed laparoscopically or with a robot, the length of stay on of and blood loss may be slightly less than with the open procedure but the long-term outcomes are similar using all three approaches. Short-term outcomes including the incidence of complications such as pancreatic fistula seem to favor laparoscopic and robotic approaches but there is no definitive data. This should be of great comfort to the patient whose only task should be to find the best (experienced) surgeon with whom they can develop a therapeutic relationship to perform the procedure at the best possible (high-volume) center in an environment where they can devote their strength to healing. Patients with malignancies of the pancreas should not be concerned about which surgical technique is used to resect their tumor.

The step-wise approach that has characterized the growth in robotic surgery of the pancreas, in contradistinction to the frenzy that accompanied the introduction of laparoscopic cholecystectomy, has allowed the identification of opportunities for improvement, many of which lie at the junction of engineering and medical practice. Improvements in robotic surgery to benefit the patient depend on a joint effort by engineers and clinicians.

## Abbreviations

DP: Distal pancreatectomy
PD: Pancreatoduodenectomy

## Author’s information

None given.
